# Factors Associated with Post-Traumatic Stress Disorder in Women Treated for Miscarriage in the Emergency Department of a Peruvian National Hospital

**DOI:** 10.3390/healthcare13172121

**Published:** 2025-08-26

**Authors:** Sofia Laura L. Zafra-Pachas, Miguel A. Arce-Huamani

**Affiliations:** 1 Facultad de Ciencias de la Salud, Universidad Privada Norbert Wiener, Lima 15046, Peru; sofialaurapachas@gmail.com; 2 Vicerrectorado de Investigación, Universidad Privada Norbert Wiener, Lima 15046, Peru

**Keywords:** spontaneous abortion, post-traumatic stress disorder, women health services, mental health, SDG 3: Good Health and Well-Being

## Abstract

Background/Objectives: Miscarriage (spontaneous abortion) can precipitate post-traumatic stress disorder (PTSD). In Peru, post-loss mental healthcare is limited. We aimed to identify factors associated with PTSD symptoms persisting ≥ 3 months among women who experienced miscarriage and were treated in the emergency department (ED) of a national hospital in Lima, 2021–2023. Methods: We conducted a cross-sectional analytical study of 214 women with spontaneous abortion seen in the ED (January 2021–December 2023). PTSD symptoms were measured with the PTSD Checklist for DSM-5 (PCL-5), anchored to the miscarriage index; sociodemographic and gyneco-obstetric variables were obtained with a validated questionnaire. Multivariable Poisson regression with robust variance estimated the adjusted prevalence ratios (aPRs). Results: Probable PTSD (PCL-5 ≥ 33) was present in 52.8% of participants. Independent correlates included previous miscarriage (aPR 1.75; 95% CI 1.35–2.25), ≥2 pre-gestational medical visits (aPR 1.66; 95% CI 1.21–2.27), and one (aPR 1.36; 95% CI 1.00–1.84) or multiple comorbidities (aPR 1.61; 95% CI 1.12–2.30). No other sociodemographic or obstetric variables were significantly associated. Conclusions: More than half of women assessed ≥ 3 months after miscarriage screened positive for probable PTSD. Previous pregnancy loss increased pre-gestational healthcare contact, and medical comorbidities were associated with higher prevalence. Integrating routine mental health screening and trauma-informed support within ED and reproductive health services could improve detection and care for this population. To our knowledge, this is the first ED-based study in Peru to examine factors associated with post-loss probable PTSD (PCL-5 ≥ 33) after miscarriage.

## 1. Introduction

Miscarriage, also referred to as spontaneous abortion, is defined as the loss of pregnancy before 20 completed weeks of gestation or the expulsion of a fetus weighing less than 500 g [1]. It is one of the most frequent adverse reproductive outcomes, with global estimates indicating that approximately 10–15% of clinically recognized pregnancies result in miscarriage [2,3]. This definition differs from induced abortion, which is the intentional termination of a pregnancy for medical or elective reasons and is not the focus of this study [4].

Although miscarriage is a biological event, it can have significant psychological consequences. One of the most studied mental health outcomes is post-traumatic stress disorder (PTSD), a condition characterized by intrusive memories, avoidance, negative alterations in cognition and mood, and hyperarousal lasting more than one month after a traumatic event [5]. The diagnostic framework provided by the Diagnostic and Statistical Manual of Mental Disorders, Fifth Edition (DSM-5), recognizes that medical emergencies, including pregnancy loss, may fulfill the criteria for a traumatic experience when associated with perceived threat to life, severe pain, or intense distress [5,6].

Previous research has reported that PTSD symptoms after miscarriage may persist for months and, in some cases, years, if left unidentified and untreated [7,8]. Risk factors include prior mental health disorders, previous pregnancy loss, low social support, and adverse obstetric experiences [8,9]. In Peru, mental health services integrated into reproductive healthcare remain limited, and there is no standardized screening for PTSD following miscarriage [10]. Evidence from other settings suggests that early identification and timely intervention can improve long-term psychological outcomes [7,9]. However, there is a lack of studies addressing this issue in the Peruvian context, particularly among women treated in emergency obstetric settings.

This study aimed to identify sociodemographic and gyneco-obstetric factors associated with PTSD symptoms persisting at least three months after miscarriage in women treated in the emergency department of a national referral hospital in Lima, Peru.

## 2. Materials and Methods

### 2.1. Study Design and Setting

We conducted an analytical cross-sectional study in the emergency department of a national referral hospital in Lima, Peru. This tertiary-level facility is a national referral center for obstetric and gynecological emergencies and serves a large and diverse urban population.

### 2.2. Population and Sample

This study included women aged 18 years or older who attended the gynecology emergency service with a confirmed diagnosis of spontaneous abortion between January 2021 and December 2023. Diagnosis was established by clinical evaluation and/or ultrasound. Eligibility required that at least three months had elapsed since the miscarriage to ensure the assessment of PTSD symptoms in the post-acute phase. Women with induced abortion, ectopic pregnancy, molar pregnancy, or incomplete medical records, as well as those who declined to participate, were excluded.

The minimum sample size was calculated using a prevalence estimate of 50% for PTSD following miscarriage to maximize variability, with a 95% confidence level and a 5% margin of error. This resulted in a required sample of 214 participants. Consecutive sampling was applied to enroll all eligible women during the study period [11].

### 2.3. Data Collection

Sociodemographic and gyneco-obstetric data were collected using a structured questionnaire adapted from prior studies on reproductive loss and mental health [8,9]. PTSD symptoms were assessed with the Posttraumatic Stress Disorder Checklist for DSM-5 (PCL-5), which consists of 20 items corresponding to DSM-5 criteria [3]. Each item is rated from 0 (“Not at all”) to 4 (“Extremely”), yielding a total score of 0–80. A cutoff score of ≥33 was used to indicate probable PTSD. PTSD symptoms were anchored to the miscarriage event (Criterion A). On the LEC-5, this corresponded to item 17 (“any other very stressful event”), following instrument guidance [12,13].

### 2.4. Variables

The dependent variable was probable PTSD (PCL-5 ≥ 33). Independent variables included age, marital status, education level, employment status, number of living children, history of previous miscarriage, gestational age at loss, number of pre-gestational check-ups (none/1/≥2; pre-pregnancy care contacts), and pre-existing medical conditions.

### 2.5. Data Analysis

Data were processed and analyzed using Stata version 17.0 (StataCorp, College Station, TX, USA). Descriptive statistics summarized the data using frequencies and percentages for categorical variables and means with standard deviations or medians with interquartile ranges for continuous variables. Associations between independent variables and PTSD were first examined with Chi-square or Fisher’s exact tests. Variables with *p* < 0.20 in bivariate analysis were included in a multivariable Poisson regression model with robust variance to estimate adjusted prevalence ratios (aPRs) and 95% confidence intervals (CIs). Statistical significance was set at *p* < 0.05.

### 2.6. Ethical Considerations

The study protocol was approved by the Ethics Committee of Universidad Privada Norbert Wiener (Approval No.: 0731-2024) and authorized by the hospital’s research committee (Letter No.: 000934-GRPA). All participants provided written informed consent prior to enrollment. This study adhered to the ethical principles of the Declaration of Helsinki.

### 2.7. Data Availability and Use of Generative AI

All data, materials, and protocols are available upon reasonable request to the corresponding author. No generative artificial intelligence (GenAI) tools were used in study design, data collection, analysis, or interpretation. GenAI was used solely for language refinement and formatting of this manuscript.

## 3. Results

### 3.1. Descriptive Analysis of Results

A total of 214 women were included. The mean age was 33.3 ± 6.1 years. Most participants were with a partner (53.7%) versus without a partner (46.3%). The majority were from Lima (81.3%); 71.0% were employed and 29.0% identified as homemakers. Regarding religion, Catholicism (74.3%) predominated, followed by other Christian denominations (25.7%). The median number of children was 1 (IQR 1–2); 50.9% reported 0–1 child and 49.1% reported 2–6 children. Most women received family support (72.4%), 18.2% received support from non-relatives, and 9.4% reported no support. Educational level was as follows: university 28.9%, technical 36.5%, and secondary 34.6%. Categorized by miscarriage type, incomplete miscarriage was most frequent (72.0%), followed by missed miscarriage (22.9%) and complete miscarriage (5.1%). The parity distribution was multiparous 53.3%, primiparous 26.2%, and nulliparous 20.6%. A previous miscarriage was reported by 47.7%. In terms of pre-gestational check-ups, 50.0% had none, 31.8% had one, and 18.2% had two or more. Regarding medical history (comorbidities), 37.9% reported none, 47.2% one, and 15.0% two or more. The median PCL-5 total score was 33 (IQR: 12–36), and 52.8% screened positive for probable PTSD (PCL-5 ≥ 33), while 47.2% did not (Table 1).

### 3.2. Bivariate Analysis

Table 2 displays the bivariate associations between clinical and sociodemographic factors and the presence of probable PTSD (PCL-5 ≥ 33). Significant associations with probable PTSD were observed for previous miscarriage (65.7% vs. 41.1%, *p* < 0.001), pre-gestational check-ups (66.7% with ≥2 check-ups vs. 44.9% with none, *p* = 0.043), and medical history (41.9% for no comorbidities vs. 57.4% for one and 65.6% for two or more, *p* = 0.034). No significant differences were observed in mean age, marital status, region of origin, occupation, religion, number of children, family support, or type of miscarriage between women with and without probable PTSD (*p* > 0.05 for all).

### 3.3. Poisson Regression Model (Multivariate Analysis)

Table 3 summarizes the crude and adjusted Poisson regression models for factors associated with probable PTSD (PCL-5 ≥ 33). After adjusting for relevant covariates, having a previous miscarriage was independently associated with a higher prevalence of probable PTSD (aPR = 1.75, 95% CI: 1.35–2.25, *p* < 0.001). Compared with women with no pre-gestational check-ups, those with two or more had a higher prevalence (aPR 1.66; 95% CI 1.21–2.27; *p* = 0.002), whereas one check-up was not significant (aPR 1.25; 95% CI 0.93–1.69; *p* = 0.123). The presence of one comorbidity (aPR 1.36; 95% CI 1.00–1.84; *p* = 0.048) and of two or more comorbidities (aPR 1.61; 95% CI 1.12–2.30; *p* = 0.009) was also associated with higher prevalence. Educational level (technical: aPR 1.44; 95% CI 1.00–2.06; *p* = 0.051; secondary: aPR 1.33; 95% CI 0.90–1.98; *p* = 0.149) and number of children (aPR 0.96; 95% CI 0.50–1.84; *p* = 0.908) were not significant. Models were adjusted for marital status, place of origin, occupation, religion, family support, educational level, miscarriage type, and parity.

## 4. Discussion

The results of this study demonstrate that probable PTSD among women treated for miscarriage is closely linked to a history of previous miscarriage, greater pre-gestational healthcare contact, and the presence of medical comorbidities. The association with prior pregnancy loss is consistent with reports showing that repeated exposure to reproductive loss can heighten psychological vulnerability and is linked to higher levels of distress and PTSD-related symptoms [14,15]. Likewise, studies in diverse settings have documented increased affective morbidity in women with prior abortions or losses, although the magnitude of association varies across populations and study designs [16,17]. Other investigations have not consistently observed this relationship, underscoring potential heterogeneity due to contextual, cultural, or methodological factors [18,19]. Nonetheless, our adjusted estimate aligns with the overall pattern indicating that a history of miscarriage is a salient risk marker for persistent post-loss symptoms [15,20].

The observed relationship between pre-gestational healthcare contact and probable PTSD may reflect underlying risk concentration. Women who require more pre-pregnancy clinical visits may have pre-existing conditions or higher perceived risk, which can amplify stress reactivity when a pregnancy loss occurs. Related evidence from maternal care pathways indicates that limited or suboptimal antenatal care is associated with greater odds of postpartum PTSD, suggesting that the organization and timing of care can influence mental health trajectories around the perinatal period [21]. Although our variable captured pre-gestational visits rather than antenatal care, the converging signal from the literature supports integrating mental health screening across reproductive care touchpoints to identify women at higher risk earlier in the care continuum [20,21].

The association between comorbid medical conditions and probable PTSD also fits with prior work showing that maternal morbidity and chronic health problems are linked to worse psychological outcomes after adverse reproductive events [21,22]. Comorbidities may increase physiological burden, healthcare utilization, and perceived vulnerability, which could sustain or intensify symptom persistence following miscarriage. While some studies have described the prevalence of medical complications without quantifying mental health associations [23], others have reported higher odds of psychopathology among women with chronic conditions or pregnancy-related disease [21,22]. Our findings expand this evidence by demonstrating an independent association in an emergency care cohort.

No significant associations were detected for sociodemographic variables after adjustment. This is consistent with several prospective and cross-sectional investigations that found limited or inconsistent effects of age, marital status, occupation, and education on post-loss psychological outcomes once clinical history and proximal stressors were considered [14,15,17,18,19,20,22]. Taken together, these patterns suggest that risk stratification may benefit most from routinely assessing reproductive history, care pathways, and comorbidity profiles rather than relying on demographic characteristics alone.

Strengths include the use of a validated instrument (PCL-5) with a commonly recommended screening threshold (≥33) and multivariable modeling to account for confounding [12,13]. The emergency department setting provides pragmatic insight into a population that is often underrepresented in mental health assessments after pregnancy loss. However, the cross-sectional design precludes causal inference and does not capture symptom trajectories over time. We did not evaluate peri-traumatic factors (e.g., the emergency care experience, perceived support during the event) that could influence symptom severity and persistence, nor did we measure potential attrition or help-seeking behaviors that might bias prevalence estimates. Our measure of “pre-gestational check-ups” may encompass heterogeneous clinical reasons and should be interpreted as a proxy for baseline healthcare contact rather than a direct measure of antenatal care intensity. Finally, reliance on self-report for PTSD symptoms, while standard in epidemiologic studies, is not a substitute for diagnostic interviewing. By design, we excluded induced abortions and anchored PTSD assessment to miscarriage; therefore, findings should not be generalized to induced abortion populations. We did not stratify results by pre-existing mental health diagnoses; although such conditions were not an exclusion criterion unless they prevented participation, residual confounding by baseline mental health cannot be ruled out.

Embedding a brief PTSD screening within reproductive health services with referral pathways for high-risk profiles prior to miscarriage, greater pre-pregnancy care contact, and medical comorbidity may improve timely identification and support. Future research should use longitudinal designs to characterize symptom resolution or persistence beyond three months, incorporate peri-traumatic and service-delivery factors, and assess whether targeted, trauma-informed interventions reduce symptom burden in this population. Beyond screening and referral, brief, structured psychological interventions have shown promise in reducing post-traumatic stress symptoms and negative affect in reproductive health contexts [24]. Although that study addressed adolescents seeking induced abortion, it underscored the feasibility of integrating time-limited, protocolized support within routine services. Adapting similar, evidence-informed modules to miscarriage care pathways and testing them in low- and middle-income country emergency settings warrants evaluation.

## 5. Conclusions

Probable PTSD is highly prevalent among women treated for miscarriage in the emergency department of a national Peruvian hospital. Independent predictors are a history of previous miscarriage, greater pre-gestational healthcare contact (≥ 2 visits), and the presence of medical comorbidities. These findings support the integration of routine PTSD screening and trauma-informed support within emergency and reproductive health services, with targeted follow-up for women presenting these risk profiles. Given the cross-sectional design, longitudinal studies are warranted to characterize symptom trajectories beyond three months and to evaluate whether timely, tailored interventions can reduce the burden of post-loss psychological morbidity.

## Figures and Tables

**Table 1:** Characteristics of women treated in the emergency department of a national referral hospital in Lima.

Characteristics	Total *n* (%)
Age	33.30 (±6.06) *
Marital status	
With partner	115 (53.74)
Without partner	99 (46.26)
Place of origin	
Lima	174 (81.31)
Outside Lima	40 (18.69)
Occupation	
Employed	152 (71.03)
Homemaker	62 (28.97)
Religion	
Catholic	159 (74.30)
Other Christian denominations	55 (25.70)
Number of children	1 (1–2)
Number of children (categorized)	
0–1 child	109 (50.93)
2–6 children	105 (49.07)
Family support	
From relatives	155 (72.43)
From non-relatives	39 (18.22)
No support	20 (9.35)
Educational level	
University	62 (28.97)
Technical	78 (36.45)
Secondary	74 (34.58)
Type of miscarriage	
Complete miscarriage	11 (5.14)
Incomplete miscarriage	154 (71.96)
Missed miscarriage	49 (22.90)
Parity	
Multiparous	114 (53.27)
Primiparous	56 (26.17)
Nulliparous	44 (20.56)
Previous abortions	
No	112 (52.34)
Yes	102 (47.66)
Pre-gestational check-ups	
None	107 (50.00)
One check-up	68 (31.78)
Two or more check-ups	39 (18.22)
Medical comorbidities	
None	81 (37.85)
One	101 (47.20)
Two or more	32 (14.95)
PCL-5 total score	33 (12–36) **
Probable PTSD	
No	101 (47.20)
Yes	113 (52.80)

* Mean ± SD; ** median (IQR).

**Table 2:** Bivariate analysis of factors associated with probable PTSD in women with miscarriages treated in the emergency department of a national referral hospital in Lima.

Characteristics	Probable PTSD		*p* Value
	No (*n* = 101) *n*%	Yes (*n* = 113) *n*%	
Age	32.83 (6.07)	33.73 (6.04)	0.278
Marital status			0.636
With partner	56 (48.70)	59 (51.30)	
Without partner	45 (45.45)	54 (54.55)	
Place of origin			0.758
Lima	83 (47.70)	91 (52.30)	
Outside Lima	18 (45.00)	22 (55.00)	
Occupation			0.325
Employed	75 (49.34)	77 (50.66)	
Homemaker	26 (41.94)	36 (58.06)	
Religion			0.989
Catholic	75 (47.17)	84 (52.83)	
Other Christian denominations	26 (47.27)	29 (52.73)	
Number of children	2 (1–2)	1 (1–2)	0.191
Number of children (categorized)			0.692
0–1 child	50 (45.87)	59 (54.13)	
2–6 children	51 (48.57)	54 (51.43)	
Family support			0.238
From relatives	71 (45.81)	84 (54.19)	
From non-relatives	17 (43.59)	22 (56.41)	
No support	13 (65.00)	7 (35.00)	
Educational level			0.115
University	36 (58.06)	26 (41.94)	
Technical	32 (41.03)	46 (58.97)	
Secondary	33 (44.59)	41 (55.41)	
Type of miscarriage			0.991
Complete miscarriage	5 (45.45)	6 (54.55)	
Incomplete miscarriage	73 (47.40)	81 (52.60)	
Missed miscarriage	23 (46.94)	26 (53.06)	
Parity			0.403
Multiparous	55 (48.25)	59 (51.75)	
Primiparous	29 (51.79)	27 (48.21)	
Nulliparous	17 (38.64)	27 (61.36)	
Previous abortion			<0.001
No	66 (58.93)	46 (41.07)	
Yes	35 (34.31)	67 (65.69)	
Pre-gestational check-ups			0.043
None	59 (55.14)	48 (44.86)	
One check-up	29 (42.65)	39 (57.35)	
Two or more check-ups	13 (33.33)	26 (66.67)	
Medical comorbidities			0.034
None	47 (58.02)	34 (41.98)	
One	43 (42.57)	58 (57.43)	
Two or more	11 (34.38)	21 (65.63)	

*p* values from χ^2^ or Fisher’s exact test for categorical variables; Student’s *t*-test for age; Mann–Whitney U for number of children. Significance set at *p* < 0.05. Source: Own elaboration.

**Table 3:** Poisson regression model of factors associated with probable PTSD in women with miscarriages treated in the emergency department of a national referral hospital in Lima.

Characteristics		Crude Analysis			Adjusted Analysis *		
		PR	95% CI	*p* Value	PR	95% CI	*p* Value
Previous abortions							
	No	Ref.			Ref.		
	Yes	1.59	1.22–2.08	<0.001	1.75	1.35–2.25	<0.001
Pre-gestational check-ups							
	None	Ref.			Ref.		
	One check-up	1.27	0.95–1.71	0.102	1.25	0.93–1.69	0.123
	Two or more check-ups	1.48	1.09–2.01	0.011	1.66	1.21–2.27	0.002
Medical comorbidities							
	None	Ref.			Ref.		
	One	1.36	1.00–1.85	0.045	1.36	1.00–1.84	0.048
	Two or more	1.56	1.09–2.23	0.015	1.61	1.12–2.30	0.009
Educational level							
	University	Ref.			Ref.		
	Technical	1.40	0.99–1.99	0.054	1.44	1.00–2.06	0.051
	Secondary	1.32	0.92–1.88	0.127	1.33	0.90–1.98	0.149
Number of children (categorized)							
	0–1 child	Ref.			Ref.		
	2–6 children	0.95	0.73–1.22	0.693	0.96	0.50–1.84	0.908

* Adjusted for the following covariates: marital status, place of origin, occupation, religion, family support, educational level, type of miscarriage, and parity; PR: prevalence ratio; 95% CI: 95% confidence interval; GLM Poisson with robust variances; source: own elaboration.

## Data Availability

De-identified data are available upon request after approval.

## Abbreviations

The following abbreviations are used in this manuscript:

PTSD	Post-traumatic stress disorder
PCL-5	PTSD Checklist for DSM-5
aPR	Adjusted prevalence ratio
CI	Confidence interval
SD	Standard deviation
IQR	Interquartile range
